# Benefits of an Exclusive Human Milk Diet in a Very Preterm Infant With an Ileostomy: A Case Report

**DOI:** 10.7759/cureus.80193

**Published:** 2025-03-07

**Authors:** Ayaka Higashizono, Masahiko Murase, Yuutarou Noguchi, Hirokazu Ikeda, Katsumi Mizuno

**Affiliations:** 1 Children Medical Center, Showa University Northern Yokohama Hospital, Yokohama, JPN; 2 Department of Pediatrics, Showa University, Tokyo, JPN

**Keywords:** abdominal surgery, case report, exclusive human milk diet, ileostomy, preterm infants

## Abstract

Mother's milk (MOM) provides numerous benefits for very preterm infants; however, it requires fortification to achieve appropriate nutrition intake. Although cow milk-based human milk fortifier (CMHMF) is widely used in Japan, it poses a risk of fatty acid calcium formation in infants with ileostomy. An exclusive human milk diet (EHMD) uses only human milk products to fortify MOM or donor human milk using a human milk-based human milk fortifier. Compared to CMHMF, EHMD reduces feeding intolerance and necrotizing enterocolitis in premature infants and may be safely used in infants with an ileostomy. However, specific studies addressing the nutritional effects of EHMD in these patients are lacking. This case report discusses the potential benefits of an EHMD in an infant undergoing ileostomy. The male infant, weighing 742 g, was delivered by emergency cesarean section at the postmenstrual age of 26 weeks and three days. On day eight of life, the patient underwent intestinal resection and anastomosis for a localized intestinal perforation. CMHMF could not be used because of the risk of fatty acid calcium stone formation. Given concerns regarding malnutrition, the case was administered an EHMD. Following the introduction of EHMD, his weight gain improved from 5 g/day to 17 g/day, and his z-score improved from -0.8 standard deviation (SD) to +0.3 SD. No adverse events were observed. It is suspected that the improvement in the anthropometric data was attributable to the establishment and maintenance of full feeding volumes alongside the EHMD, which ensured adequate nutrition and increased nutritional intake. This case suggests that EHMD may be a safe and effective nutritional strategy to promote optimal growth and development in infants undergoing abdominal surgery.

## Introduction

Mother’s milk (MOM) provides numerous health benefits to infants with very low birth weight (VLBW). It contains essential macronutrients, micronutrients, and bioactive components, such as immunoglobulins, cytokines, growth factors, and antimicrobial agents. Studies have shown that higher doses of MOM are associated with reduced incidences of necrotizing enterocolitis (NEC), late-onset sepsis, chronic lung disease, retinopathy of prematurity, and neurodevelopmental impairment [1]; therefore, donor human milk (DHM) is recommended when MOM is unavailable [2].

Full-term infants can get sufficient nutrition from human milk alone. However, fortifiers are needed because preterm infants require more protein and other nutrients than full-term infants to ensure appropriate growth and brain development [3,4]. Thus, multi-nutrient fortifiers can be added to human milk to meet nutritional standards [5]. A cow milk-based human milk fortifier (CMHMF) is widely used in Japan; however, it may cause fatty acid calcium stones in infants with ileostomies [6]. At our institution, we avoid CMHMF in VLBW infants following abdominal surgery, though this typically results in extrauterine growth restriction.

An exclusive human milk diet (EHMD) is defined as the use of only human milk products by fortifying MOM or DHM with a human milk-based human milk fortifier (HMHMF; Prolacta Bioscience, California, USA). EHMD for premature infants reduces feeding intolerance, NEC incidence, and hospitalization costs compared with milk-based diets [7-9]. EHMD may be used to provide adequate nutrition to infants who undergo abdominal surgery without adverse effects. To the best of our knowledge, no previous study has specifically assessed the nutritional influence of EHMD on body weight gain in VLBW infants following abdominal surgery.

We present this case to provide insights into the potential benefits of EHMD in VLBW infants. Written informed consent was obtained from the patient’s legal guardians for the publication of this case report.

## Case presentation

**Figure 1:**
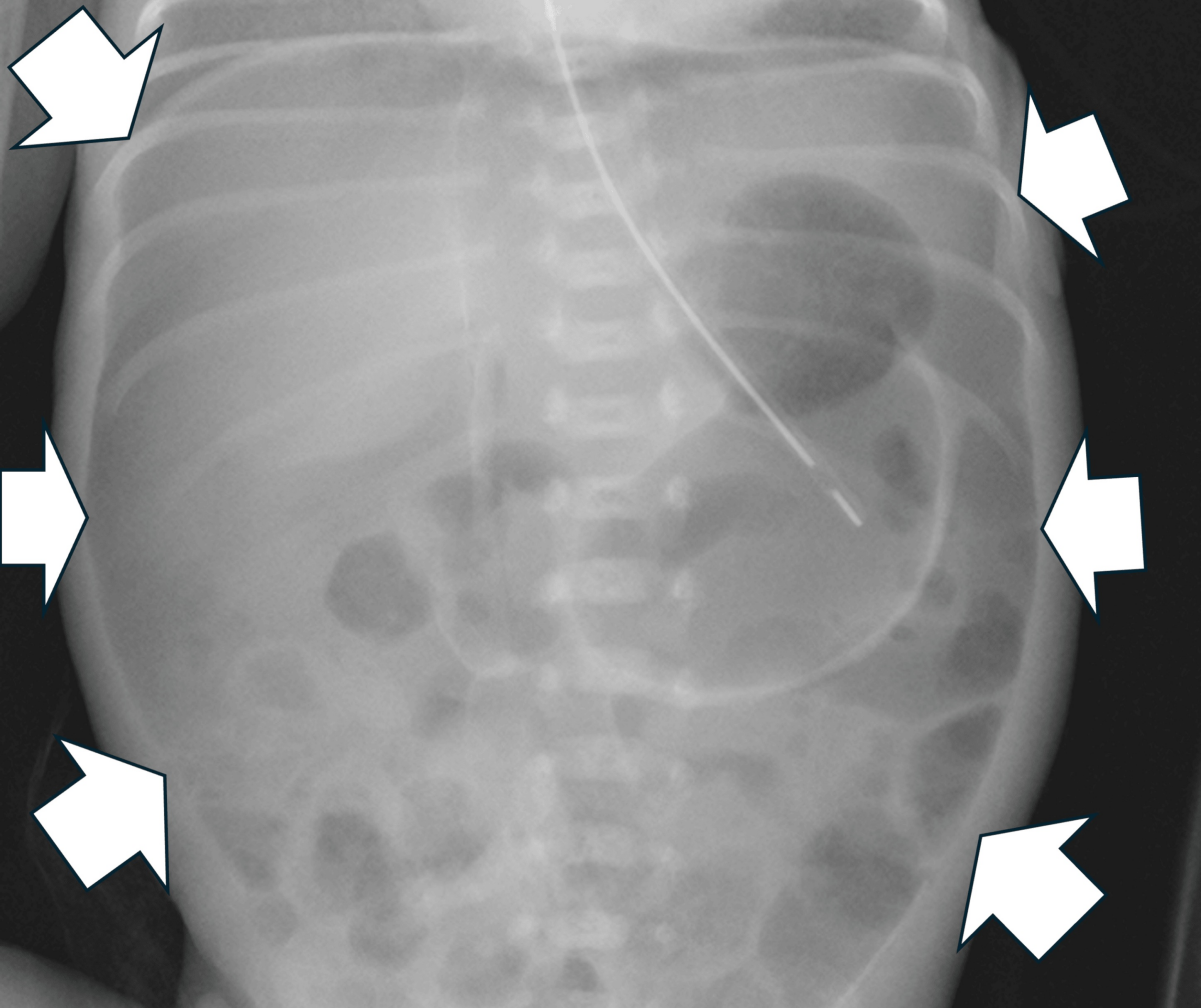
Abdominal radiograph finding in the supine position on day of life eight. Figure shows the air dome sign on a supine (white arrow).

**Figure 2:**
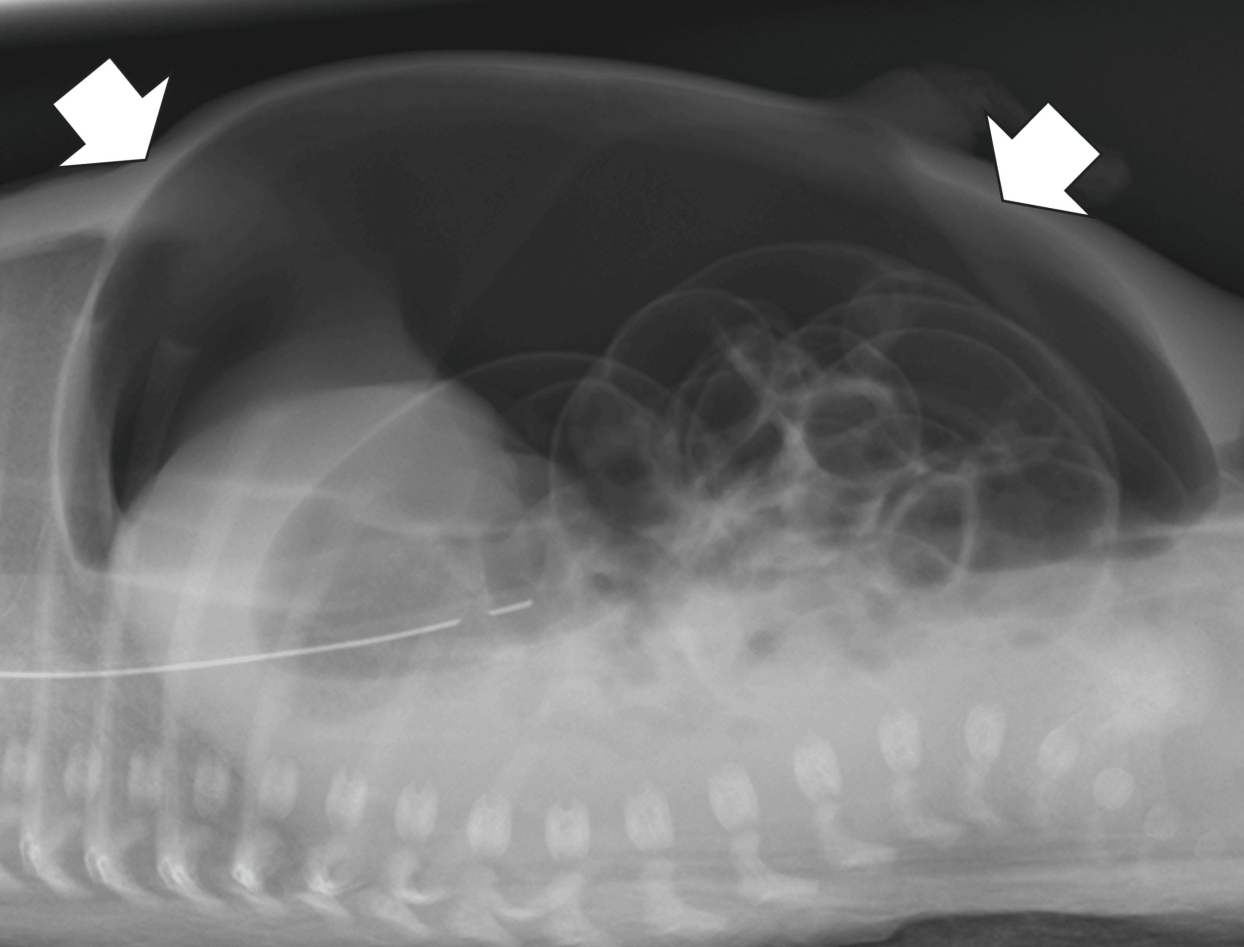
Abdominal radiograph finding in the cross-table lateral view position on day of life eight. Figure shows free air on a supine cross-table lateral view (white arrow).

A male infant weighing 742 g was delivered by emergency cesarean section at a postmenstrual age of 26 weeks and three days due to superimposed preeclampsia. His Apgar scores were 4 and 7 at 1 min and 5 min, respectively. Intubation was initiated immediately after birth due to the lack of spontaneous respiration. Surfactants were administered due to respiratory distress syndrome. Enteral feeding with donor human milk was initiated on day two of life (DOL 2) because MOM could not be obtained on the same day. The patient developed abdominal distension on DOL 6. The blood test results showed white blood cell count (WBC), 6910/μL and C-reactive protein (CRP) level, 2.58 mg/dL; the X-ray findings did not show abdominal free air. Therefore, NEC development was assumed; however, he did not develop perforation. He was subsequently administered ampicillin sodium and gentamicin sulfate, wherein enteral nutrition was discontinued as non-surgical management. However, because his abdominal distension gradually worsened, he underwent abdominal radiography on DOL 8, which revealed free air (Figures 1, 2). He was diagnosed with gastrointestinal perforation and underwent abdominal surgery that day. A 1.5 cm perforation from the ileum was found at the terminal 8.5 cm site. Intraoperative gross and pathological findings of the intestine did not show necrotizing findings; the patient was diagnosed with focal intestinal perforation (FIP). Subsequently, resection and direct anastomosis were performed.

Since we did not use cow milk-based human milk fortifier (CMHMF) in this patient because of the risk of fatty acid calcium stones, the patient was suspected of developing growth failure due to malnutrition. Thus, we determined that the patient required an EHMD. Although it is Food Drug Administration-approved for use in VLBW infants, its use in Japan is considered off-label. Since approval from our hospital’s committee for evaluating unapproved or off-labeled medicinal products and medical devices was required to use HMHMF in our patient, we applied to our hospital’s committee for evaluating unapproved new drugs. 

**Figure 3:**
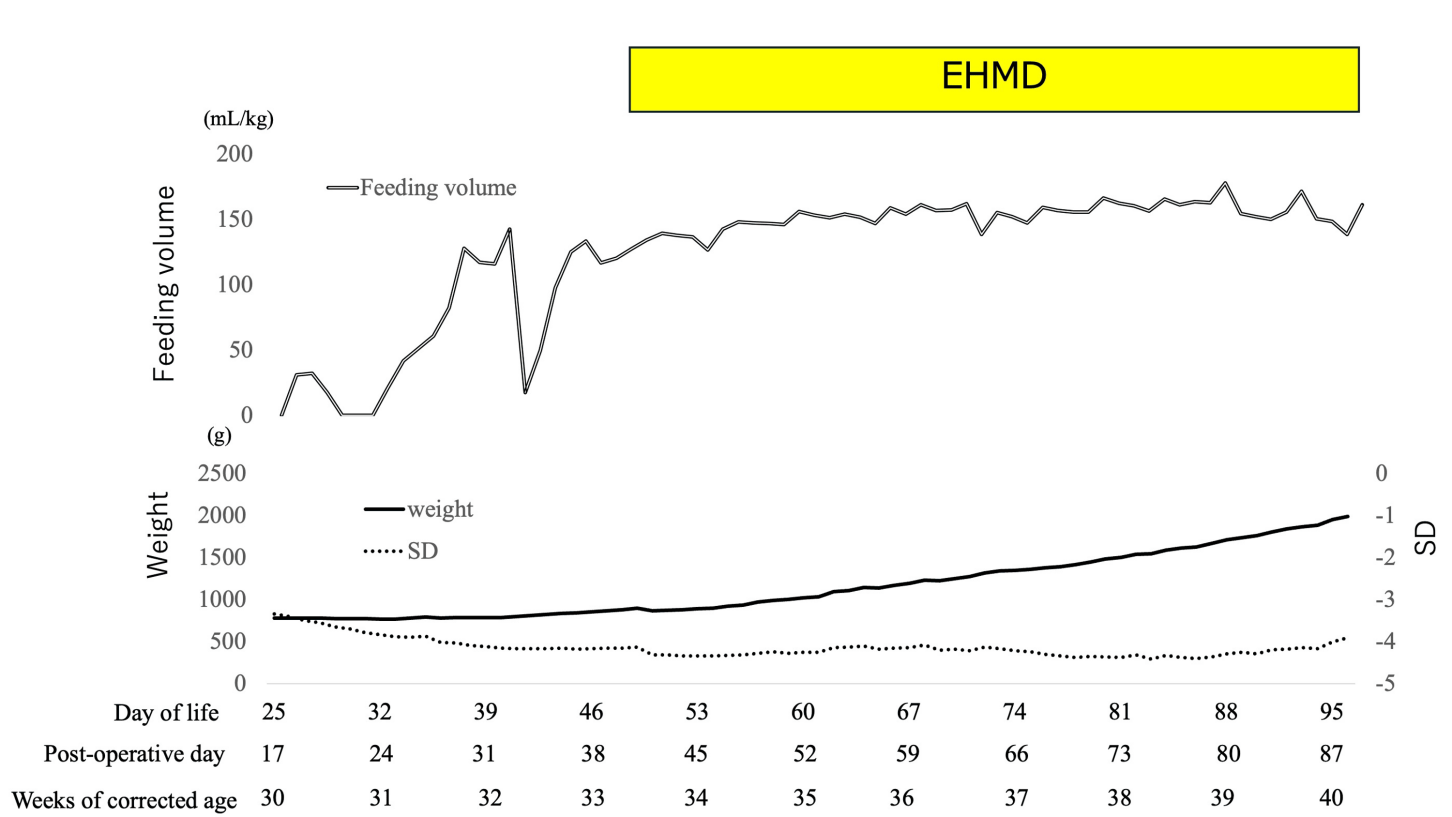
The association between weight, SD, and feeding volume before and during the exclusive human milk diet (EHMD). The graph illustrates the body weight (solid line), SD (dot line), and feeding volume (double line). The EHMD was used from the day of life (DOL) 48 to 96. Weight gain and SD improved after the EHMD, and adequate milk volume and nutrition were obtained.

Enteral feeding was started on DOL 20; however, the patient could not initially increase his feeding volume due to feeding intolerance. Figure 3 shows the weight and weight standard deviation (SD) trends, enteral feeding volume, and fortification schedule. The patient’s condition was critically exacerbated, wherein we could not measure his weight until DOL 25 (the initiation day in Figure 3 was DOL 25 and 30 weeks of corrected age). Finally, the feeding volume gradually increased from DOL 32, which was achieved at 140 mL/kg/day on DOL 40. However, since gastric residue increased on DOL 41, enteral feeding was stopped on the same day. As the gastric residual improved, the enteral feeding was restarted at 50 mL/kg/day on DOL 42. Since there was no gastric residual recurrence, feeding volume gradually increased. We received approval from our hospital’s committee for evaluating unapproved or off-labeled medicinal products. His feeding volume was achieved at 127mL/kg/day, without abdominal symptoms. Thus, medical HMHMF was initiated for use on DOL 48 (33 weeks and two days of corrected age). We obtained consent from his family to use HMHMF with the document form and initiated HMHMF half-strength on DOL 48 and increased it to full strength on DOL 51, adding cream from DOL 53. The patient achieved full enteral feeding on DOL 54, wherein EHMD was terminated on DOL 96 (40 weeks and one day of corrected age). 

**Figure 4:**
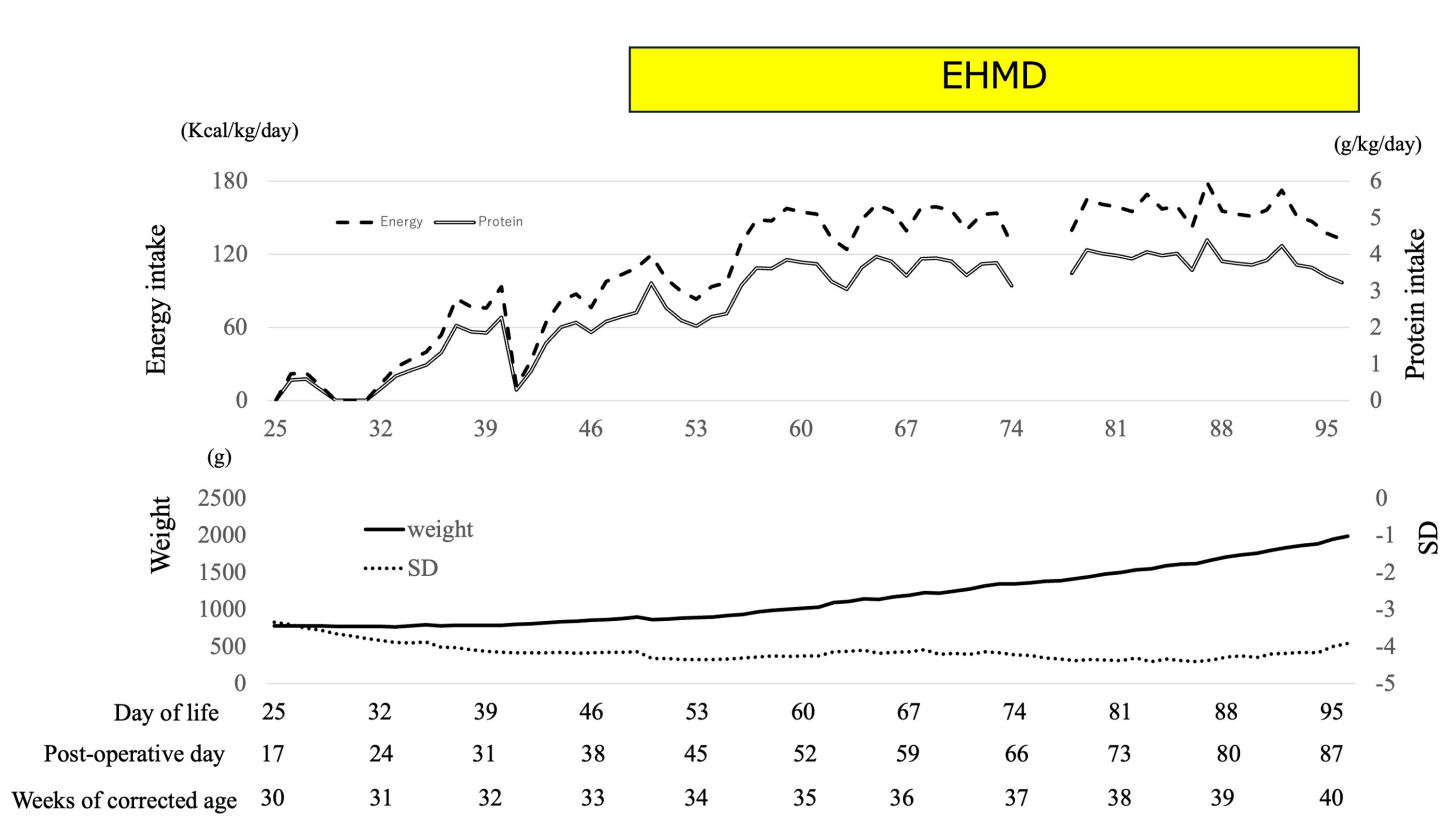
The association between weight, SD, energy, and protein intake before and during the exclusive human milk diet (EHMD). The graph illustrates the body weight (solid line), SD (dot line), energy intake (dashed line), and protein intake (double line). The EHMD was used from the day of life (DOL) 48 to 96. Weight gain and SD improved after the EHMD, and adequate nutrition was obtained. The DHM administered from DOL 75 to 77 did not have nutritional data, and we could not calculate the nutritional data these days. Thus, lines were missing these days.

Figure 4 shows the weight and weight standard deviation (SD) trends, energy, protein intake, and the fortification schedule. DHM content was analyzed using data from the Human Milk Bank Association. Since the MOM volume was low, DHM was used, as in a previous study [10]. Since the DHM administered from DOL 75 to 77 did not have nutritional data, we could not calculate the nutritional data these days. This case developed skin erosion due to malnutrition, wherein a peripherally inserted central catheter could not be kept. This case also did not receive parental nutrition. Since this case received low calories via parental, we only calculated the nutritional data via enteral nutrition. Increased nutritional intake has been associated with improved weight gain. This case used almost EHMD administration during the period of time of using HMHMF because the artificial formula was administered from DOL 74 and DOL 75 due to a shortage of DHM and lack of MOM. No gastrointestinal symptoms associated with formula use were observed during the study period. 

Table 1 compares anthropometric data, nutritional intake, and laboratory data before and after EHMD. Body weight growth velocity was calculated using established formulas [11]; SD was calculated using the Japanese standard [12]. Before and after using EHMD, all factors were increased.

**Table 1:** The comparison of anthropometric data, nutritional intake, and laboratory data before and after EHMD. EHMD: exclusive human milk diet, SD: standard deviation.

	Before using EHMD	After using EHMD
Anthropometric data		
Body weight growth velocity (g/kg/day)	5	18
Weight SD change	–0.8	0.3
Nutritional data		
Average feeding volume (mL/kg)	65	152
Average protein intake (g/kg/day)	1.1	3.5
Average energy intake (kcal/kg/day)	44	142
Laboratory data		
Albumin (g/dL)	2.8	3.2
Pre-albumin (mg/dL)	3.6	5.9

## Discussion

We report a case wherein we implemented an EHMD to improve the anthropometric data. This effect is attributed to establishing and maintaining full feeding volumes alongside the EHMD and increasing nutrition. Due to EHMD intake, CMHMF was not used. As a result, the full feeding volume was maintained, contributing to weight gain. The EHMD is associated with a shorter duration of feeding intolerance than CMHMF [13]. It is possible that this patient did not have feeding intolerance after achieving full feeding due to EHMD intake. In addition, EHMD has been linked to a lower risk of NEC compared with CMHMF [14]. This lower risk allowed for a safe increase in the feeding volume, further contributing to the improvement in anthropometric data. 

In this case, EHMD intake provided adequate nutrition; therefore, the infant exhibited improved weight gain and serum albumin levels. Previous reports have shown that the protein content of DHM is significantly lower than that of preterm human milk [15]. The donor milk used in this case also has nutritional components similar to those reported in previous studies, and it has low protein content compared with the mature human milk composition reported in previous studies [10,15]. Consequently, infants receiving DHM are at higher risk of growth failure than those receiving MOM. Although formula feeding can result in higher short-term growth rates, it poses an increased risk for NEC [4]. Therefore, EHMD serves as a crucial treatment option for fulfilling the nutritional needs of infants while minimizing the associated risks. 

Since the artificial formula had to be used for two days in this case, this case was administered almost EHMD during the period of time of using HMHMF. The shortage of donor milk was caused by inadequate inventory management; unexpected increases in milk consumption due to infant weight gain and feeding volume increments, combined with weekend delivery suspension, resulting in a shortage. 

Although EHMD intake improved the anthropometric data, its long-term effect on neurodevelopment remains unclear. Thus, an extended follow-up is needed. A limitation of this study is that since the composition of human milk was not directly determined, the reported nutritional values were theoretical estimates. Consequently, the actual quantity of nutrients administered to the infants might differ. 

EHMD was used, in this case, until 40 weeks and one day of corrected age. Since HMHMF is prescribed as off-label use in Japan, in this case, it might have been stopped earlier. However, since there is no established duration for using EHMD in cases of ileostomy, we discussed about how long to use EHMD in this baby and decided on this period of time of use for the following reasons. EHMD was initiated from 33 weeks and two days of corrected age. If EHMD was only used during the preterm period, this case could intake it for less than four weeks; we thought that this was a short period of time. In addition, our case exhibited a weight of 1346 g at 37 weeks 0d day of corrected age, wherein severe malnutrition was developed. If EHMD was stopped and CMHMF was initiated, malnutrition would have worsened due to the risk of fatty acid calcium salt formation.

The case used preterm formula without adverse events, wherein short-term use was well-tolerated. However, long-term use might develop feeding intolerance. Since the case stopped feeding and developed more severe malnutrition due to feeding intolerance, we did not prescribe EHMD after preterm formula use. Finally, the case’s weight increased to 1988 g on the last day of EHMD and 40 weeks one-day gestation without any adverse events. EHMD could decrease hospitalization costs and improve feeding intolerance [9], and reduce the cost without adverse events. However, this case might have used EHMD for unnecessary periods of time and achieved a weight increase due to this duration. The period of time of using EHMD must be considered on an individual case-by-case basis, as this case of ileostomy with EHMD intake is the first to be reported to date. When the period of using the EHMD is decided, considering weight is more appropriate compared with corrected age because growth is essential. When more cases are reported, the appropriate period of time of using EHMD will be established based on its merits, rate of complications, and costs.

## Conclusions

We report an almost EHMD administration during the period of time of HMHMF use in a VLBW infant with an ileostomy at our hospital. The EHMD administration improved weight gain and SD changes. The improvement in the anthropometric data might be attributable to the establishment and maintenance of full feeding volumes alongside the EHMD use, which ensured adequate nutrition and increased nutritional intake without observing adverse events. This case report highlighted the potential for the safe and effective use of EHMD in cases of abdominal surgery.
